# Feasibility of an Audit and Feedback Intervention to Facilitate Journal Policy Change Towards Greater Promotion of Transparency and Openness in Sports Science Research

**DOI:** 10.1186/s40798-022-00496-x

**Published:** 2022-08-06

**Authors:** Harrison J. Hansford, Aidan G. Cashin, Matthew K. Bagg, Michael A. Wewege, Michael C. Ferraro, Sina Kianersi, Evan Mayo-Wilson, Sean P. Grant, Elaine Toomey, Ian W. Skinner, James H. McAuley, Hopin Lee, Matthew D. Jones

**Affiliations:** 1 School of Health Sciences, Faculty of Medicine and Health, UNSW Sydney, Sydney, Australia; 2 Centre for Pain IMPACT, Neuroscience Research Australia, Sydney, Australia; 3 Faculty of Health Sciences, Curtin Health Innovation Research Institute, Curtin University, Perth, Australia; 4 Perron Institute for Neurological and Translational Science, Perth, Australia; 5 Department of Epidemiology and Biostatistics, Indiana University School of Public Health-Bloomington, Bloomington, IN USA; 6 Department of Social & Behavioural Sciences, Indiana University Richard M, Fairbanks School of Public Health at Indianapolis, Indianapolis, IN USA; 7 Health Research Institute School of Allied Health, University of Limerick, Limerick, Ireland; 8 School of Allied Health, Charles Sturt University, Exercise and Sport Sciences, Port Macquarie, Australia; 9 Centre for Statistics in Medicine, Nuffield Department of Orthopaedics, Rheumatology and Musculoskeletal Sciences, University of Oxford, Oxford, UK; 10 School of Medicine and Public Health, University of Newcastle, Newcastle, Australia

**Keywords:** Open science, Transparency, Replicability, Sports science

## Abstract

**Objectives:**

To evaluate (1) the feasibility of an audit-feedback intervention to facilitate sports science journal policy change, (2) the reliability of the Transparency of Research Underpinning Social Intervention Tiers (TRUST) policy evaluation form, and (3) the extent to which policies of sports science journals support transparent and open research practices.

**Methods:**

We conducted a cross-sectional, audit-feedback, feasibility study of transparency and openness standards of the top 38 sports science journals by impact factor. The TRUST form was used to evaluate journal policies support for transparent and open research practices. Feedback was provided to journal editors in the format of a tailored letter. Inter-rater reliability and agreement of the TRUST form was assessed using intraclass correlation coefficients and the standard error of measurement, respectively. Time-based criteria, fidelity of intervention delivery and qualitative feedback were used to determine feasibility.

**Results:**

The audit-feedback intervention was feasible based on the time taken to rate journals and provide tailored feedback. The mean (SD) score on the TRUST form (range 0–27) was 2.05 (1.99), reflecting low engagement with transparent and open practices. Inter-rater reliability of the overall score of the TRUST form was moderate [ICC (2,1) = 0.68 (95% CI 0.55–0.79)], with standard error of measurement of 1.17. However, some individual items had poor reliability.

**Conclusion:**

Policies of the top 38 sports science journals have potential for improved support for transparent and open research practices. The feasible audit-feedback intervention developed here warrants large-scale evaluation as a means to facilitate change in journal policies.

*Registration*: OSF (https://osf.io/d2t4s/).

**Supplementary Information:**

The online version contains supplementary material available at 10.1186/s40798-022-00496-x.

## Key Points

- An audit-feedback intervention to facilitate policy change in sports science journals appears feasible to conduct on a larger scale.
- The TRUST form used in our audit-feedback intervention, based upon the TOP guidelines, has moderate reliability.
- The top 38 sports science journals have potential for improved support for transparent and open research practices.

## Background

Transparent and open research practices are fundamental for research findings to be critiqued and evaluated, reproduced or replicated, and to inform clinical or policy decisions [1]. However, such practices are yet to be widely adopted by researchers or encouraged by journals, funders, and institutions [2, 3]. Many researchers have raised concerns at the alarming number of studies that have failed to reproduce (i.e. independent researchers analysing the same data and getting the same result) or replicate (i.e. independent researchers collecting new data, analysing it and getting the same result) the findings of other researchers [4, 5]. This ‘reproducibility crisis’ [4] has been identified in psychology [6], social science [7], neuroscience [8], biomedicine [9], and is speculated to be present in sports science, with several recent calls for change [10–12]. There are emerging efforts to improve transparency and openness in sports science research [10–12], including the establishment of initiatives such as the Society for Transparency, Openness and Replication in Kinesiology and the Consortium for Transparency in Exercise Science [12]. The challenges to transparent and open research are systematic and cultural [13], and will require coordinated efforts between research stakeholders to drive change [14, 15].

Scientific journals disseminate the vast majority of scientific literature [16]. There are many important roles within a journal that impact journal policy, including editor(s)-in-chief, editorial boards, publishers and affiliated research societies. The editor(s)-in-chief of many journals are key research stakeholders because of their role as the ‘gatekeeper’ of the scientific findings they choose to publish. Journals’ editorial policies can influence the reporting of research. For example, changes in these policies have been associated with improved registration and reporting of randomised controlled trials [17–21]. It is therefore plausible that interventions targeting the transparency and openness editorial policies of journals (for example, via feedback to the editor/s-in-chief) might in turn improve the transparency and openness of the research they publish.

The Transparency and Openness Promotion (TOP) guidelines [22] (Box 1) were created by research stakeholders to articulate standards for transparent and open research practices. The TOP guidelines also function as a framework for journals to improve the transparency and openness of research they publish; through expression of standards for transparent and open practices in their editorial policies. The extent to which journal policies adhere to these standards have been evaluated in several fields: pain [3], sleep [23] and social intervention research [24]. This work indicates poor overall expression of the standards. However, interventions targeted at improved expression of these standards in journal editorial policies have rarely been evaluated and reported publicly [3]. Prior to evaluating the effectiveness of an intervention to improve promotion of transparent and open research practices of journals, it is important to understand whether such an intervention is feasible (i.e. can it be done, should we proceed with it, and how?) [25, 26].

**Box 1:** TOP Guidelines

TOP Standard	Description	Proposed Benefit
Data and Code Citation	Data and code citation involve the appropriate citation of pre-existing data and code used in the study	Appropriate citation of data and code helps recognize and credit these as original intellectual contributions
Data; Analytic Methods (code); and Research Materials Transparency	Transparency of Data, code and research materials involves a statement indicating availability of these materials, and providing direction to them. This can be facilitated by public repositories such as OSF (osf.io), FigShare (figshare.com) and Dryad (datadryad.org)	Statements around, and the sharing of data, code and research materials aid the evaluation, verification or reproduction of research, as well as aiding evidence synthesis
Design and Analysis Transparency	Design and analysis transparency involve appropriately reporting all aspects of research, facilitated by adhering to relevant reporting guidelines where available	Standards for reporting research design and analysis should maximize transparency about the research process and minimize potential for vague or incomplete reporting of the methodology. This may improve clinical translation of evidence, replication and proper evaluation
Preregistration of Studies	Preregistration of studies involves a statement whether the study was preregistered, and information on how to access it	Study preregistration is very important to reduce questionable research practices, allow readers to assess any deviations from the pre-planned study and increase visibility of the research even if the study does not get published
Preregistration of Analysis Plans	Preregistration of analysis plans involves an indication whether or not the conducted research was preregistered with an analysis plan, including the sequence of analyses or statistical model that will be reported	Preregistration of Analysis Plans certifies the distinction between confirmatory and exploratory research
Replication	While not formally a transparency standard for authors, this section addresses journal policy for consideration of independent replications for publication, i.e. whether the journal encourages submission of replication studies or Registered Reports	Allowing replication studies to be published promotes verification and increased confidence in results

The primary aim of this study was to evaluate the feasibility of an audit-feedback intervention designed to facilitate sports science journal policy change towards greater promotion of transparency and openness. We also aimed to evaluate the reliability of a tool that assesses journal support for open science practices and to evaluate the degree to which the policies of leading sports science journals currently support transparent and open research practices. The effectiveness of our audit-feedback intervention on changing journal policies was not an aim of this feasibility study.

## Methods

This study was prospectively registered on the *Open Science Framework* (OSF) (https://osf.io/ceb8u/). All data, code and materials supporting the findings are available on the OSF repository (https://osf.io/d2t4s/). Journal TOP Factor scores are available at topfactor.org (https://topfactor.org/journals?disciplines=Sports+Science) to enable comparison of Sports Science to other disciplines.

### Design

This study comprised a feasibility assessment of an audit-feedback intervention targeted at journals’ editorial policies, an evaluation of the reliability of a tool for this purpose, and a cross-sectional audit of transparency and openness standards in sports science journals’ editorial policies (Additional file 1). We reported reliability in accordance with the Guidelines for Reporting Reliability and Agreement Studies (GRRAS) [27].

### Outcomes

Our predefined criteria for feasibility of the audit-feedback intervention were the time taken to (1) rate each journal’s policies, and (2) format and submit individualised letters to the editor. The audit-feedback intervention was regarded as feasible if, on average, it took less than 30 min to rate the journal’s policies and less than 45 min to create and submit the tailored letter to the editor. We established additional feasibility outcomes post hoc*,* including: qualitative feedback from raters, qualitative acceptability to journal editors (i.e. how well our feedback was received), and fidelity of the intervention delivery (i.e. how well we were able to deliver all letters). Outcomes for reliability were inter-rater reliability and absolute agreement of the Transparency of Research Underpinning Social Intervention Tiers (TRUST) policy evaluation form (hereafter TRUST form). Outcomes related to journal support for transparent and open research practices were each journal’s TRUST form score (individual items and overall), as well as requirements for disclosures of conflicts of interest. The effectiveness of our audit-feedback intervention on changing journal policies was not an outcome of interest for this feasibility study.

### Outcome Measures

The TRUST form [24], based upon the TOP Factor (a metric of the degree to which journals comply with the TOP guidelines) [28], was used to audit the editorial policies of sports science journals. This form included items based on standards in the TOP Guidelines [22] and an additional indicator of whether journals offer Registered Reports as a publication type. All items are scored on a 0–3 scale; where Level 0 indicates that the journal does not implement the standard and Level 3 indicates that the journal requires and verifies the standard. Variants of this form have been used in prior work [29, 30]. The ICMJE Form for Disclosure of Potential Conflicts of Interest [31] was used to evaluate the conflict of interest requirements for each journal. Adherence to each standard (Additional file 1) was measured on a 0 to 4 scale with a score of zero indicating the journal policy made no statement of the standards and a score of four indicating statement of all standards or requirement that authors submit the ICMJE disclosure form. The TRUST form was implemented with Research Electronic Data Capture (REDCap) [32, 33] (See https://osf.io/d2t4s/ for REDCap Codebook). We determined relative inter-rater reliability using the intraclass correlation coefficient for agreement (ICC (2,1)) and absolute reliability using the standard error of measurement; for individual TOP Factor items and the total TOP Factor score using the TRUST form. The reliability strata were < 0.5 = poor, 0.5–0.75 = moderate, 0.75–0.9 = good, > 0.9 = excellent [34]. We did not assess inter-rater reliability or agreement for the items that could be skipped based on answers to preceding questions (i.e. secondary items that may not be displayed for all raters or journals). Post-hoc, we calculated the smallest detectable change to determine a ‘real’ change beyond measurement error (i.e., a change beyond a threshold created from a distribution of change scores that one would expect if measurement error was the only source of variance for the instrument) [35].

### Sample

The sample size calculation was based on the reliability for the overall score of the TRUST form between raters. We required 38 journals to detect good reliability (ICC = 0.8) between the three raters with a 95% confidence interval of 0.7–0.9 [36]. We consecutively sampled the first 38 sports science journals by impact factor (identified using the “Sports Science” filter on Web of Science) [37] (Table 1).

**Table 1:** Scores for the top 38 sports science journals on the TRUST form and ICMJE requirement for disclosure of conflict of interests

Journal Name	Citation		Transparency				Registration		Replication	Overall TRUST score	ICMJE disclosure score
	Data	Code	Data	Code	Material	Design & analysis	Analysis plan	Study			
1. British Journal of Sports Medicine	0	0	1	0	0	2	0	0	0	3	4
2. Sports Medicine	0	0	1	1	0	1	0	1	0	4	2
3. American Journal of Sports Medicine	0	0	0	0	0	2	0	0	0	2	4
4. Exercise Immunology Review	0	0	0	0	0	0	0	0	0	0	0
5. Journal of Sport and Health Science	0	0	0	0	0	1	0	0	0	1	4
6. Journal of The International Society of Sports Nutrition	0	0	1	1	1	1	0	1	0	5	4
7. Exercise and Sport Sciences Reviews	0	0	0	0	0	0	0	0	0	0	4
8. Arthroscopy—The Journal of Arthroscopic and Related Surgery	0	0	0	0	0	1	0	2	0	3	4
9. Medicine and Science in Sports and Exercise	0	0	0	0	0	0	0	0	0	0	4
10. International Journal of Sport Nutrition and Exercise Metabolism	0	0	0	0	0	1	0	0	0	1	0
11. Journal of Orthopaedic & Sports Physical Therapy	0	0	1	0	0	2	0	2	0	5	3
12. Journal of Science and Medicine in Sport	0	0	0	0	0	2	0	2	0	4	4
13. International Journal of Sports Physiology and Performance	0	0	0	0	0	0	0	0	0	0	3
14. Scandinavian Journal of Medicine & Science in Sports	0	0	1	0	0	1	0	0	0	2	4
15. Knee Surgery Sports Traumatology Arthroscopy	0	0	1	1	1	1	0	3	0	7	4
16. Clinical Journal of Sport Medicine	0	0	1	0	0	1	0	0	0	2	4
17. Archives of Physical Medicine and Rehabilitation	0	0	0	0	0	2	0	0	0	2	4
18. Journal of Applied Physiology	0	0	2	0	0	1	0	0	0	3	0
19. Journal of Strength and Conditioning Research	0	0	0	0	0	0	0	0	0	0	1
20. Sports Health—A Multidisciplinary Approach	0	0	0	0	0	0	0	1	0	1	4
21. Quest	0	0	0	0	0	0	0	0	0	0	4
22. Psychology of Sport and Exercise	0	0	0	0	0	2	0	0	1	3	4
23. Journal of Shoulder and Elbow Surgery	0	0	0	0	0	1	0	0	0	1	2
24. European Journal of Sport Science	0	0	0	0	0	0	0	0	0	0	4
25. Sport Education and Society	0	0	0	0	0	0	0	0	0	0	4
26. Sociology of Sport Journal	0	0	0	0	0	0	0	0	0	0	0
27. Journal of Sports Sciences	0	0	1	0	0	0	0	3	0	4	4
28. European Journal of Applied Physiology	0	0	1	0	0	1	0	3	0	5	4
29. International Journal of Sports Medicine	0	0	0	0	0	0	0	0	1	1	1
30. Research in Sports Medicine	0	0	1	0	0	0	0	0	0	1	4
31. Applied Physiology Nutrition and Metabolism	0	0	1	0	0	1	0	3	0	5	4
32. Orthopaedic Journal of Sports Medicine	0	0	0	0	0	2	0	3	0	5	4
33. Journal of Athletic Training	0	0	0	0	0	1	0	3	0	4	2
34. Journal of Sport Management	0	0	0	0	0	0	0	0	0	0	0
35. Gait & Posture	0	0	0	0	0	1	0	2	0	3	4
36. Journal of Sport & Exercise Psychology	0	0	0	0	0	0	0	0	0	0	0
37. Clinics in Sports Medicine	0	0	0	0	0	0	0	0	0	0	2
38. Journal of Applied Sport Psychology	0	0	1	0	0	0	0	0	0	1	4

### Audit

We considered the journals’ policies regarding transparent and open practices and their requirements for disclosing conflicts of interest to be reflected in the ‘guidance’ or ‘instructions to authors.’ We employed a 2-click rule for locating author guidelines on the journal’s home or linked web-pages. If the instructions provided a link to the broader policies of the journals publishing house (e.g., Taylor & Francis or Elsevier), these were also assessed. One author (HJH) sourced the online journal ‘guidance/instructions to authors’ or the equivalent section of the respective journal websites on 24 May 2021. The same author saved the relevant web page(s) in HTML format and used Apple Preview (OSX 11.5.2) to create time-stamped, PDF files. Journal policies were rated independently by at least three authors (i.e. in triplicate) from a pool of five authors (AGC, HJH, MAW, MCF, MDJ). Disputes were resolved with recourse to an author not involved in the triplicate rating. These authors were PhD Candidates, early-mid career researchers and an honours student, some having experience in conducting a similar audit.

### Intervention

The behaviour our audit-feedback intervention sought to change was adherence to transparency and openness standards within journal editorial policies. We intervened at the level of the journal editor-in-chief. We used the data describing the journal’s support for transparent and open research practices and requirements for disclosing conflicts of interest to construct a tailored letter for each editor-in-chief. This letter informed the editor-in-chief of their journal’s scoring and comparison with the other 37 journals. Each letter also contained individualised information describing the importance of improving adherence to the recommended transparency standards (see Additional file 1, for template). To ensure consistency in implementation of the intervention, the tailored letter was, where possible, electronically submitted as a letter to each journal using the respective submission portals. Where journals did not accept letters, we had to modify our mode of delivery, with the letter instead emailed to the journal’s editor/s-in-chief using a standardised template (Additional file 1).

### Analysis

Quantitative data were analysed with R (version 4.0.2) [38–43] and Statistical Product and Service Solutions (SPSS) [44]. Feasibility was analysed as the time taken (total and average per journal) to rate journal policies and submit the tailored letters. Editorial receipt of the letter was verified through publication, rejection or email response. We assessed relative and absolute reliability of the TRUST form using an intraclass correlation coefficient (two-way random effects, absolute agreement, multiple raters model (ICC (2,1)) [45] (Eq. 1) and the standard error of measurement (Eq. 2), respectively.

1 $$ICC\left(\mathrm{2,1}\right)= \frac{{s}_{r}^{2}}{{s}_{r}^{2} + {s}_{c}^{2} +{s}_{residual}^{2}}$$

ICC(2,1) formula, S^2^r = variance of subjects, S^2^c = variance of bias from raters, s^2^residual = random error variance.

We calculated the standard error of measurement using an agreement formula that was analogous to the ICC model and included systematic differences between raters in the calculation.

2 $$SEM agreement= \sqrt{{s}_{t}^{2} + {s}^{2}residual}$$

Equation 2: Standard error of measurement. SEM = standard error of measurement, s_t_^2^ = variance due to systematic differences between raters, s^2^residual = random error variance.

Inter-rater reliability was reported with 95% confidence intervals (CI). The 95% CI’s were calculated from the *psych* R package [39] in accordance with Shrout and Fleiss, 1979 [45]. We calculated the smallest detectable change using an established formula using the standard error of measurement for agreement [46]. We summarised journal scores as median and range due to the non-normal distribution of the data, and all other continuous data with mean (SD).

### Methodological Differences to the Protocol

In order to improve the interpretability of our findings we calculated the smallest detectable change of the TRUST form in addition to the standard error of measurement and intraclass correlation coefficient. We planned to include 36 journals in our audit-feedback intervention. However, during the course of our study we became aware that two journals on our list were already being evaluated in a separate but related study [24]. To avoid intervening on these journals twice, we did not include them in our audit-feedback intervention but still included them in our reliability evaluation. Therefore, we added an additional two journals, bringing the total sample to 38 (n = 36 included in the audit-feedback intervention, n = 38 included in the reliability analysis).

## Results

### Feasibility

It took 2 h total to locate and download the ‘guidance’ forms for all 38 journals. It took 17.4 h to rate all journals in triplicate, an average of 9 (5) minutes for each rater per journal. It took 18.4 h total to prepare and submit the letters; an average of 29 (10) minutes per letter.

From the letters submitted through formal publication pathways (n = 15), all were confirmed as received by editors. In contrast, of those emailed (n = 16), only two were confirmed to be received. We were unable to submit six letters due to those journals not accepting letters to the editor and being unable to locate the email of the journal editor/s (e.g. the editor was a practicing clinician and had no publicly available email address). We did not submit a letter to one journal as they had been included in a previous assessment using the TRUST form [24]. Regarding fidelity of the intervention delivery, 17 editors acknowledged receipt of the letter (n = 2, published [47, 48]; n = 1, invited editorial [49]; n = 12, rejected; n = 2, email discussion). When ordered by impact factor, only six journal editors (33%) in the top 19 journals did not acknowledge the letter, whereas 14 editors (78%) in the bottom 19 journals did not acknowledge the letter. At the time of submission, approximately 6 months after the letters were submitted, we are still awaiting response from 14 (39%) journal editors. Therefore, we cannot ascertain receipt of the letter to these editors.

### Qualitative Feedback from Raters and Journal Editors

The raters highlighted differences in formatting ‘author guidelines’ between journals as the primary challenge when rating journals. These between-journal differences increased the time taken to find and score each journal policy. Nine editors who rejected the letter for publication responded by email stating they would discuss the findings with their editorial board. One editor suggested immediate changes to their policies whereas another editor was unaware of the TOP guidelines. One editor reported the open science practices supported by the journal were decided by the publisher and two editors expressed that time to change policies was a barrier for editorial staff.

### Relative and Absolute Reliability of the TRUST Form

The overall relative inter-rater reliability was moderate [34] [ICC (2,1) = 0.68 (95% CI 0.55–0.79)] with a standard error of measurement of 1.17. The relative reliability of individual items ranged from 0 to 1. Several items (1a, 1b, 2a, 3a, 5a, 9c) had poor reliability (ICC < 0.5) (Table 2). Data for inter-rater reliability and standard error of measurement for each item of the TRUST form, and the overall score, are shown in Table 2. The smallest detectable change of the overall TRUST form was 3.2.

**Table 2:** Inter-rater reliability and overall agreement of the TRUST form

Item name	Relative inter-rater reliability (intraclass correlation coefficient (2,1))	Absolute reliability (standard error of measurement)
1a. Data citation	0.49 [0.33 to 0.64]	0.31/1
1b. Code citation	0.00 [− 0.17 to 0.18]	0.09/1
2a. Data transparency	0.00 [− 0.13 to 0.39]	0.23/1
3a. Code transparency	0.00 [− 0.14 to 0.18]	0.09/1
5a. Research material transparency	0.23 [0.07 to 0.42]	0.16/1
6a. Design and analysis	0.86 [0.80 to 0.91]	0.19/1
7a. Study registration	0.78 [0.69 to 0.86]	0.23/1
8a. Registration of analysis plan	*	*
9a. Acceptance of replication studies	0.80 [0.70 to 0.87]	0.09/1
9b. Registered Reports	1.00 [1.00 to 1.00]	0.00/1
9c. Submission of background and methods alone	0.00 [− 0.14 to 0.18]	0.09/1
Overall	0.68 [0.55 to 0.79]	1.17/27

*No variance in ratings (all 38 journals received a “No” for this item), therefore an intraclass correlation coefficient and standard error of measurement could not be determined

### Journal Policies Transparency and Openness Scores

**Fig. 1:**
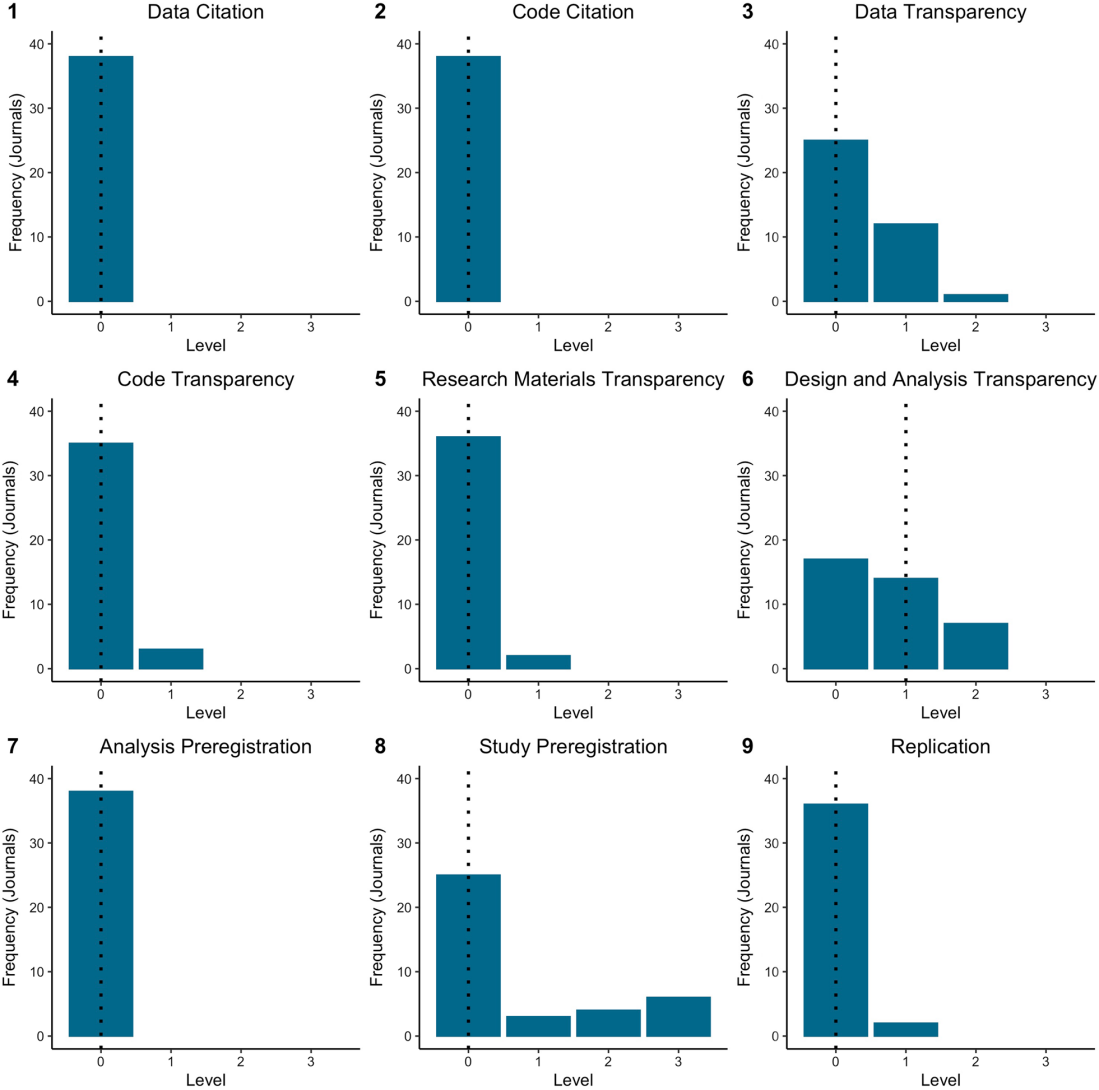
Summary of scores for each item of the TRUST form. The dotted line indicates the median score for each item. For TOP items 1–8, a score of: 0 = not mentioned or ‘encouraged’ by the journal policy (not implemented), 1 = statement regarding standard required by the journal policy (disclosed), 2 = adherence to standard required by the journal policy (require), 3 = required and verified by the journal policy (verify). For item 9, a score of: 0 = not mentioned by the journal policy, 1 = journal states significance or novelty are not criteria for publication, 2 = journal reviews replication studies blinded to results, 3 = journal accepts registered reports

**Fig. 2:**
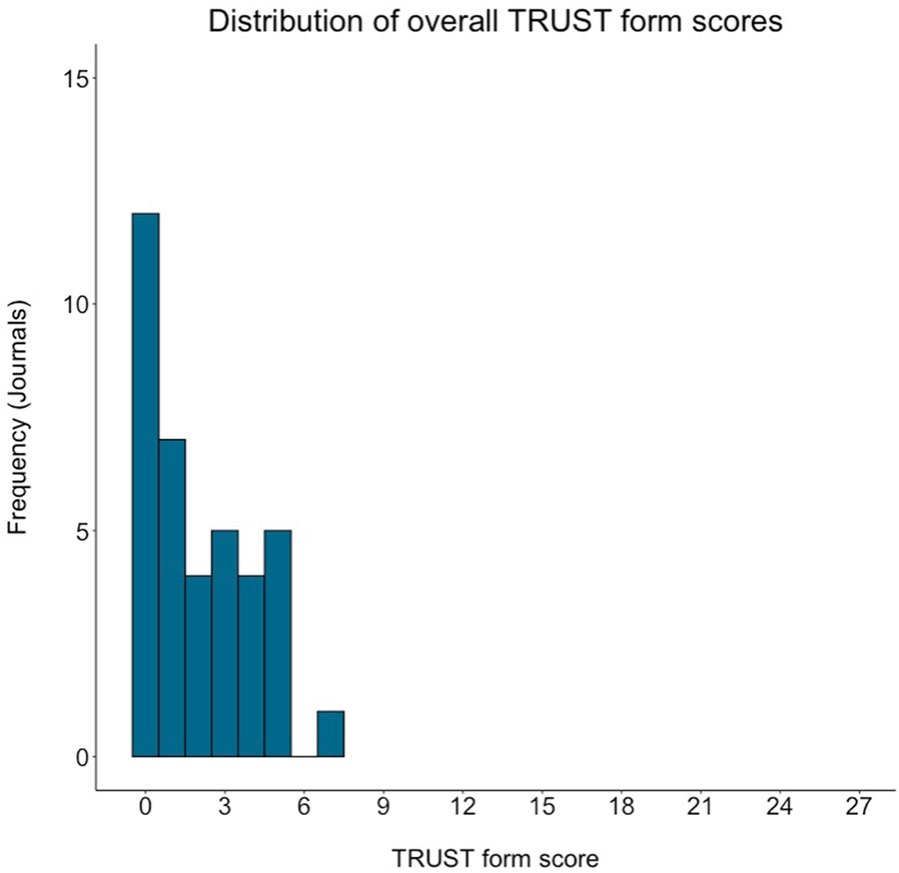
Distribution of the overall TRUST form scores from each journal (range = 0 to 27)

**Fig. 3:**
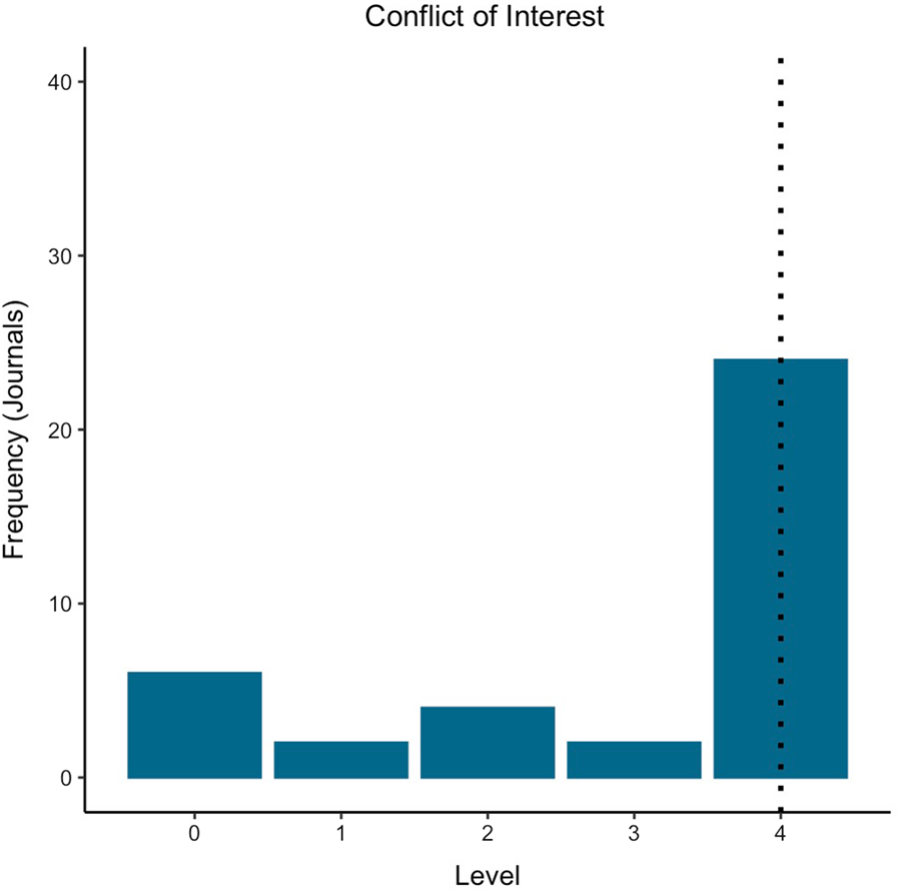
Summary of score for adherence to the ICMJE Form for Disclosure of Potential Conflicts of Interest. The dotted line indicates the median score

Table 1 contains the TRUST form and ICMJE disclose of conflicts of interest scores for all 38 journals. All journals scored 0 for the *data citation, code citation,* and *analysis transparency* standards. The *data transparency, code transparency, materials transparency* and *replication* standards all had a median score of 0 (range 0–1). The *study preregistration* standard had a median score of 0 (range 0–3), and the median score of the *design and analysis transparency* standard was 1 (range 0–2) (Fig. 1). The mean (SD) score (0–27) on the TRUST form across all 38 journals was 2.05 (1.99) (Fig. 2). The highest score was 7 and the lowest score was 0. The mean disclosure of conflicts of interest score (0–4) for all 38 journals was 2.95 (1.56) (Fig. 3).

## Discussion

We evaluated the feasibility of an audit-feedback intervention designed to facilitate policy change in the leading sports science journals towards greater adherence to transparency and openness standards. We found our intervention was feasible and that the TRUST form had moderate reliability, implying suitability of our approach for use in a future randomised controlled trial aiming to change journal policy. Our evaluation of the sports science journal’s engagement with transparency and openness standards indicated substantial room for improvement. In contrast, the journals demonstrated high engagement with standards for disclosure of conflicts of interest.

It has been suggested that feasibility studies should not evaluate the effectiveness of interventions, but aid in the decision to conduct a larger study, and guide its development [25]. Based on our predefined, time-based criteria [50], our intervention was feasible. However, feasibility can extend beyond just the time taken to perform the intervention. For example, the mode of delivery of the intervention can impact its fidelity, and thus its feasibility. We are still awaiting a response from 14 journals (38% of the sample) regarding our letter. The high proportion of non-response suggests another method of delivery or form of intervention may be required for a future randomised controlled trial evaluating the effectiveness of our intervention to change journal policy [51]. Furthermore, qualitative feedback from raters indicated the process was laborious, therefore performing the intervention on a larger scale or within a trial, may require a larger team or a change in approach. For example, two rather than three raters could rate each journal, reducing the workload for each rater and increasing the number of journals rated in a given time. Refinement of the audit-feedback intervention is warranted before it is rigorously tested in a randomised controlled trial. Given the low scores of all journals on the TOP Guidelines, however, we believe an intervention is needed, and a randomised controlled trial evaluating the effectiveness of our audit-feedback intervention on changing journal policy may be one such approach. For example, by randomising journals to receive feedback or not and then comparing their policies after an appropriate time-period (noting that journal policy change may take months-years to occur). We acknowledge there may be issues with this approach, such as when publishers, not journals, are responsible for setting journal policies (as noted by feedback received from one Editor-in-chief in our study). This issue would require careful consideration in a randomised controlled trial but nonetheless, we believe such a trial is warranted.

Proper assessment of how journal policies promote transparency and openness is an important first step in changing such policies. Proper assessment relies on tools that are valid and reliable. Determination of validity requires comparison to a gold standard (criterion validity) or to a similar tool that assesses the same construct (construct validity). While tools have been developed to assess some transparency and openness indicators [52], the TOP Guidelines were developed by experts and are considered the gold standard for transparency and openness. Therefore, their construct validity in measuring journals’ openness and transparency policies is assumed. We note however, that the TOP Guidelines are not without criticism nor universally supported [53]. For example, the effectiveness of the TOP Guidelines for improving transparency and openness has been questioned, as has the evidence to support the inclusion of each item in the Guidelines [53]. Our results show that the TRUST form tool has moderate relative reliability overall, but poor reliability for some individual items (1a, 1b, 2a, 3a, 5a, 9c) (Table 3). However, due to the lack of variance (i.e., > 95% same value) exhibited in some of these items (1b, 3a, 9c), the low ICC value may be more reflective of this lack of variation rather than poor relative reliability of the tool. Indeed, the interaction between measurement error and natural variation forms the basis of reliability and agreement [35]. Thus, with minimal residual variation, an item may have poor relative reliability even with a relatively small measurement error, as there is inadequate variation to distinguish journals. This was observed in some of the abovementioned items in which journals scoring poorly on those questions, or similarly, the requirements of the TOP Factor, the basis of the TRUST form, was too stringent for these items, reducing variation between journals. The former is more likely, as other journals that have been assessed with the tool have attained higher and more varied scores [54]. Several items had poor relative reliability, it is therefore important that the overall score from the TRUST form is used to assess journal openness and transparency, as this allows adequate variability to distinguish between journals.

The utility of a measurement instrument extends beyond simply its relative reliability. For example, the standard error of measurement and the smallest detectable change (i.e., change beyond measurement error) are two important measures that should be considered when determining the potential utility of the TRUST form. While there is no clear guidance on interpreting the standard error of measurement, the value found here for the TRUST form of 1.17 (out of a total of 27) can be considered small measurement error, indicating good absolute reliability. The smallest detectable change of the overall TRUST form was 3.2, indicating that changes in scores greater than this could be distinguished from measurement error and considered ‘true’ change. Therefore, if journals made changes to their policies relating to the TOP Guidelines, even if only to improve their scores by one level on four items, the tool would be able to detect this change. We believe the low values for standard error of measurement and smallest detectable change indicate the TRUST form is appropriate to assess change in journal policy over time. For example, in a randomised controlled trial of an intervention targeted at improving expression of the TOP guidelines in journal editorial policies.

Identifying shortcomings of journal policies in the field of sports science is a necessary first step toward improving the transparency and openness of sports science research. We showed that the top 38 sports science journals scored poorly on all domains of the TRUST form. Almost every TOP standard had a median score of 0 (not implemented), with only *design and analysis transparency* having a median score of 1 (disclosed) (Table 2). This poor engagement with open science practices at the journal policy level is similar to other fields [3] and presents ample opportunity for sports science journals to revise their policies to improve the transparency and openness of sports science research. The TOP Guidelines [55] provide recommendations on how journals can modify their policies to improve the reproducibility and replicability of research they publish.

Qualitative feedback provided by some editors highlighted a willingness to improve their journal’s policies but identified time and other logistical concerns as barriers to this. Adoption of the level 1 TOP guidelines may be a suitable starting point to address these concerns because this level was designed to have minimal impact on editorial workload while making measurable inroads to improving transparency and openness [55]. For example, level 1 requires articles to provide statements describing whether data, code, research materials, analysis plans and study preregistrations are publicly accessible, and providing guidance on data and code citation while not requiring authors to provide such materials or verification [55]. These changes can be made with relatively little disruption to existing workflows and would likely having a meaningful impact on improving the reproducibility of sports science research. Further guidance on how journal policies can be modified to improve transparency and openness are provided by the Centre for Open Science (osf.io/kgnva/).

Conflicts of interest can unintentionally influence research design, conduct and reporting. Thus, disclosure of conflicts of interest are important to reduce bias and increase confidence in science [56]. Most sports science journal policies required all four ICMJE conflict of interest standards, with an overall mean of 2.95/4, similar to other fields [3, 57] (Fig. 3). We would encourage journals who did not require all conflict of interest standards to consider revisiting their policies to assess whether requiring the ICMJE form (or similar) at submission would improve trust that conflicts are transparently reported.

Our study is not without limitation. First, we only used the TRUST form to evaluate the promotion of transparency and openness of journals. The standards in the TOP Factor and TRUST form may not completely capture all the ways a journal could promote transparent and open research, for example, publishing open access research. Secondly, we assessed the journals based on the information presented on their website. We assumed this information would reflect journal policies at the time of assessing publications, but this may not be the case. For example, the website for *Exercise Immunology Review* was undergoing maintenance, so a cached version of the website from 2020 was sourced. Furthermore, we were unable to completely assess fidelity of the intervention delivery because we were unable to confirm receipt of the letter to 14 (39%) journal editors. This may have limited the ability for feedback to be received by editors, a consideration for audit-feedback approaches in future studies. Finally, due to the low number of journals that scored > 1 on any item, the ability of the TRUST form to reliably distinguish between higher levels remains unknown. That is, we can currently distinguish with moderate reliability between journals that have a policy compared to those that do not. However, we are unsure whether we can reliably distinguish between journals that have a “lenient” policy compared to those that have a more “stringent” policy. Limited inter-rater reliability may also reflect ambiguities in journal policies whereby we expect these instruments would be more reliable if instructions to authors were clearer. Assessment of the TRUST form reliability in disciplines which have a wider range of TOP Factor scores (e.g., medicine [58]) is need to better understand the relative reliability of the tool [24] and its suitability for future use in a randomised controlled trial evaluating change in journal policy.” Limited inter-rater reliability may also reflect ambiguities in journal policies. That is, we expect that these instruments would be more reliable if instructions to authors were clearer.

## Conclusions

Transparency and openness in science promotes reproducibility, replicability and ultimately trust in research findings. The TRUST form is reliable and can feasibly be used as part of an audit-feedback intervention to rate journal policies on a larger scale. Our audit of the top 38 sports science journals’ policies shows significant room for improvement in the requirement of open science practices. Journals can improve the transparency and openness of research in the field by adopting policies that facilitate greater transparency and openness. Minimally resource-intensive audit-feedback interventions may provide one potential avenue towards helping facilitate these practices.

## Supplementary Information

**Additional file 1**.** Supplementary Material 1**. Letter template.** Supplementary Material 2**. ICMJE disclosure of conflict of interest form and levels.** Supplementary Material 3**. Email template to journal editors.

## Data Availability

All quantitative data and code are publicly accessible (https://osf.io/d2t4s/).
