# Supplementary material for: Feasibility of an Audit and Feedback Intervention to Facilitate Journal Policy Change Towards Greater Promotion of Transparency and Openness in Sports Science Research
Source: Sports Med Open. 2022 Aug 6;8:101. doi: 10.1186/s40798-022-00496-x (PMC9357245; doi:10.1186/s40798-022-00496-x)
Supplement: Supplementary file 1 — Additional file 1. Supplementary Material 1. Letter template. Supplementary Material 2. ICMJE disclosure of conflict of interest form and levels. Supplementary Material 3. Email template to journal editors. [file 40798_2022_496_MOESM1_ESM.docx]

**Supplementary Material**

**Supplementary Material 1:** Letter template

Dear ,

Editor-in-chief, [journal name]

Increased transparency and openness of sport science research will improve the ability to appraise, replicate, and implement research findings. However, sport science journals need to support and prioritise practices that bolster transparency and openness to facilitate this (1,2). Journal policies that actively support transparency and openness have been shown to improve the quality, reproducibility and replicability of research (3). To date, over 1,100 journals and funding agencies have ascribed to implementing one or more of the eight standards for transparent and open research described in the Transparency and Openness Promotion (TOP) Guidelines (4).

We report an appraisal of how well the policies of the leading 38 sport science journals support transparent and open research practices. The [journal name] was evaluated using the TOP Factor (https://osf.io/t2yu5/) – an alternative metric for evaluating the degree to which journal policies promote transparency and openness (5), and the International Committee of Medical Journal Editors (ICMJE) disclosure of interest requirements (6). The TOP Factor is calculated as the sum of journal implementation of the eight modular standards within the TOP Guidelines and an additional standard related to publication of replication studies. The requirements for disclosure of conflicts of interest were evaluated using the four standards stated in the ICMJE disclosure form (6). The [journal name]’s TOP Factor and conflict of interest scores are provided in Figures 1 and 2 respectively, alongside summary data from the top 38 sport science journals.

[Insert Figure 1]

Figure 1. Summary of scores for each item of the TOP Factor. The dotted line indicates the [journal name]’s score for each item, with the colour indicating the position relative to the median score of the top 38 sports science journals. Black indicates the score is equal to the median, red indicates the score is below the median (worse), and green indicates the score is above the median (better). For TOP items 1-8, a score of: 0 = not mentioned or ‘encouraged’ by the journal policy, 1 = recommended by the journal policy, 2 = required by the journal policy, 3 = required and enforced by the journal policy. For item 9, a score of: 0 = not mentioned by the journal policy, 1 = journal states significance or novelty are not criteria for publication, 2 = journal reviews replication studies blinded to results, 3 = journal accepts registered reports.

[Insert Figure 2]

Figure 2. Summary of score for adherence to the ICMJE form for Disclosure of Potential Conflicts of Interest. The dotted line indicates the location of the [journal name]’s score, with the colour indicating the position relative to the mean score of the top 38 sports science journals. Black indicates the score is equal to the mean, red indicates the score is below the mean (worse), and green indicates the score is above the mean (better).

Briefly, the [journal name] scored well for TOP Factor items “data transparency”, “design and analysis transparency”, “study preregistration”, poorly for items “data citation”, “code citation”, “code transparency”, “research materials transparency”, “analysis preregistration”, “replication”, and had a total TOP Factor score of XX / 27 (higher scores represent increased requirement for transparency and openness by the journal). The [journal name] was above/below the mean TOP Factor score of 2.05 ± 1.99 / 27, and was above/below the mean ICMJE disclosure of conflicts score (2.95 ± 1.56 / 4) compared to the top 38 sports science journals.

We strongly encourage the [journal name] to review and, where necessary, update your journal’s policies (see https://cos.io/top/ for advice and resources to implement this). Greater transparency and openness will improve the conduct and translation of sport science research to ultimately move the field forward. Moreover, complete and transparent disclosure of conflicts of interest will increase confidence in the research findings. By publishing this letter, the [journal name] is taking action towards improving the transparency and openness of sports science research.

References

1. Ioannidis, J.P. How to make more published research true. Plos Medicine 2, e1001747

(2014).

2. Munafò, M.R., Nosek, B.A., Bishop, D.V.M., Button, K.S., Chambers, C.D., Percie du Sert, N., et al. A manifesto for reproducible science. Nature Human Behaviour 1, 0021 (2017).

3. Cashin, A.G., Bagg, M.K., Richards, G.C., Toomey, E., McAuley, J.H. & Lee, H. Limited engagement with transparent and open science standards in the policies of pain journals: a cross-sectional evaluation. BMJ evidence-based medicine (2020).

4. Nosek, B.A., Alter, G., Banks, G.C., Borsboom, D., Bowman, S.D., Breckler, S.J., et al. Promoting an open research culture. Science 348, 1422-1425 (2015).

5. Mayo-Wilson, E., Grant, S., Supplee, L., Kianersi, S., Amin, A., DeHaven, A., et al. Evaluating implementation of the Transparency and Openness Promotion (TOP) guidelines: the TRUST process for rating journal policies, procedures, and practices. Research Integrity and Peer Review 6, 9 (2021).

6. International Commitee of Medical Journal Editors. Conflicts of interest. http://www.icmje.org/conflicts-ofinterest/.

**Supplementary Material 2**: ICMJE disclosure of conflict of interest form and levels

*ICMJE Disclosure of Potential Conflicts of Interest Standards*

1. Recipient of payment or services from a third party (government, commercial, private foundation, etc) for any aspect of the submitted work (including but not limited to grants, data monitoring board, study design, manuscript preparation, statistical analysis, etc);
2. Any financial relationships (regardless of amount of compensation) with entities in the bio-medical arena that could be perceived to influence, or that give the appearance of influencing, the submitted work;
3. Any patents, whether planned, pending or issued, broadly relevant to the submitted work and
4. Any other relationships or activities that readers could perceive to have influenced, or that give the appearance of potentially influencing, the submitted work.

*ICMJE Form for Disclosure of Potential Conflicts of Interest*

The following 4 sections from the ICMJE form for disclosure of potential conflicts of interest were used as a “gold standard” to measure journal adherence:

1. The work under consideration for publication.
   1. Did you or your institution at any time receive payment or services from a third party (government, commercial, private foundation, etc.) for any
   2. aspect of the submitted work (including but not limited to grants, data monitoring board, study design, manuscript preparation, statistical analysis,
   3. etc.)?
2. Relevant financial activities outside the submitted work.
   1. Indicate whether you have financial relationships (regardless of amount of compensation) with entities as described in the instructions.
3. Intellectual Property.
   1. Do you have any patents, whether planned, pending or issued, broadly relevant to the work?
4. Relationships not covered above
   1. Are there other relationships or activities that readers could perceive to have influenced, or that give the appearance of potentially influencing what you wrote in the submitted work

**Supplementary Material 3**: Email template to journal editors

Dear *[journal name]* Editorial Office and [Editor in Chief/s],

Our team has appraised the journal policies of the 38 leading sports science journals regarding their support for transparent and open science practices. The *[journal name]* was included in this appraisal.

Please find attached a summary of the findings from this evaluation as well as guidance on how the *[journal name]* can better support transparent and open science practices.

We hope you acknowledge these findings and consider updating the *[journal name]’s* policies to ultimately improve the transparency and openness of published sports science research.

Kind Regards,

Harrison Hansford, on behalf of the TOP Sports Science team
